# Efficacy and Effectiveness Outcomes of Treatments for Double‐Exposed Chronic Lymphocytic Leukemia and Small Lymphocytic Lymphoma Patients: A Systematic Literature Review

**DOI:** 10.1002/cam4.70258

**Published:** 2024-09-30

**Authors:** Mohammed Zuber, Sreelatha Akkala, Niying Li, Sajesh K. Veettil, Chia Jie Tan, Lorenzo Villa Zapata

**Affiliations:** ^1^ Clinical and Administrative Pharmacy, College of Pharmacy University of Georgia Athens Georgia USA; ^2^ School of Public Health The University of Texas Health Science Center at Houston Houston Texas USA; ^3^ Department of Pharmacy Practice, School of Pharmacy IMU University Kuala Lumpur Malaysia; ^4^ School of Medicine Taylor's University Subang Jaya Selangor Malaysia; ^5^ Department of Pharmacotherapy, College of Pharmacy University of Utah Salt Lake City Utah USA

**Keywords:** BTKi, chronic lymphocytic leukemia, double exposed, double refractory, efficacy, relapsed/refractory, systematic review

## Abstract

**Background:**

Bruton's tyrosine kinase inhibitors (BTKi) and the B‐cell lymphoma 2 (BCL2) inhibitor venetoclax have significantly improved outcomes and achieved durable remission in patients with chronic lymphocytic leukemia (CLL). BTKi/venetoclax‐treated patients with exposure to both novel agents (regardless of the reason for discontinuation) are classified as “double‐exposed,” and often have poor prognoses. This study aims to assess the efficacy and effectiveness of treatments in double‐exposed CLL patients.

**Methods:**

PubMed, Embase, and Web of Science databases were searched until December 2023.

**Results:**

We retrieved 3948 articles for screening and included 13 publications covering nine distinct studies. Three clinical trials reported a median PFS of 16.8 months with pirtobrutinib, 13 months with lisocabtagene maraleucel, and 10.1 months with nemtabrutnib. ORR ranged from 58% with nemtabrutinib and 80% with lisocabtagene maraleucel. In observational studies, PFS ranged from 3 months with chemoimmunotherapy to 12 months with BTKi, and ORR ranged from 31.8% with chemoimmunotherapy to 85.7% with chimeric antigen receptors (CAR) T‐cell therapy.

**Conclusion:**

This study highlights the limited clinical data on efficacy outcomes for double‐exposed CLL/SLL patients. Pirtobrutinib, lisocabtagene maraleucel, and a combination of ibrutinib and venetoclax have shown promising effects. However, the scarcity of treatment options and efficacy data for patients who have failed BTKi and venetoclax underscores a significant unmet medical need.

## 1 Introduction

Chronic lymphocytic leukemia (CLL) is a lymphoproliferative disorder, characterized by the proliferation and accumulation of mature B‐cell lymphocytes that are immunologically defective [1]. In 2024, the estimated new cases of CLL in the United States were 20,700 (12,690 in males and 8010 in females), and the estimated deaths were 4440 (2790 males and 1650 females) [2]. Bruton tyrosine kinase inhibitors (BTKi) and venetoclax are relatively novel agents that are important components of treatment regimens used to treat both newly diagnosed and relapsed/refractory CLL. While most patients achieve a durable response, these treatments are not curative and disease progression can occur [3, 4]. The choice of treatment regimen in the relapsed refractory setting is determined by an understanding of previous lines of treatment, the reason for prior treatment discontinuation (progression or toxicity), and the mechanisms of resistance to treatments [5].

Following the approval of ibrutinib, a first‐in‐class covalent BTKi, the treatment landscape in CLL has evolved rapidly, particularly over the past decade [6]. Acalabrutinib and zanubrutinib are two additional covalent BTKi that have received approval after ibrutinib [7, 8]. Both have demonstrated comparatively better efficacy and safety than ibrutinib in relapsed or refractory settings [8, 9, 10]. Covalent (irreversible) BTKi form a covalent bond, which attaches to the cysteine 481 (C481) residue of BTK, thereby effectively obstructing the ATP‐binding pocket and halting its catalytic function. However, despite favorable results with these treatments in CLL patients, resistance often develops over time in a significant number of cases caused by the mutation at the C481 residue [11, 12, 13].

Venetoclax, an orally available small molecule inhibitor, selectively targets BCL‐2. It is approved for use in front‐line and relapsed/refractory settings, both in combination with anti‐CD20 monoclonal antibodies or as monotherapy, and in patients with del (17)(p13.1) CLL who have received at least one previous line of treatment [14]. The combination of ibrutinib and venetoclax has shown promising progression‐free survival (PFS) and response rates in treatment naïve CLL patients who are older and/or those with comorbidity. However, venetoclax poses a risk of rapid tumor lysis syndrome, necessitating careful dose escalation and inpatient monitoring for certain patients [15].

Evidence from a retrospective study of 10 US academic centers showed that 51% of patients discontinued ibrutinib due to toxicity, 28% due to progression, 8% due to Richter's Transformation (RT), and 11% due to unrelated death/ other reasons [16]. Furthermore, first‐line venetoclax discontinuation was 40%, as reported in a retrospective study that utilized the Flatiron database [17]. The most common reasons for venetoclax discontinuation were CLL progression (35.3%), toxicity (19.3%), planned cellular therapy (14.3%), and RT (10.9%) [17]. According to Aronson et al., 2022, BTKi/venetoclax‐treated patients with exposure to both novel agents (regardless of the reason for discontinuation) are classified as “double‐exposed” patients [18]. Whereas, patients who are exposed to both a BTKi and venetoclax and who are believed to be resistant to both classes of agents are defined as “double refractory” patients [18]. Following the discontinuation of covalent BTKi and venetoclax, the outcomes in CLL patients are extremely poor, with less than 6 months median time to subsequent treatment failure or death [19, 20]. Overall, very limited data exist on the efficacy outcomes in double‐exposed patients.

To our knowledge, a systematic review has not been conducted on available treatments and their efficacy and effectiveness outcomes for double‐exposed CLL patients. Therefore, we have aimed to conduct a systematic review to synthesize evidence from both clinical trials and real‐world settings on the treatments and their efficacy/effectiveness in double‐exposed CLL patients. This study will help inform clinicians on various treatments used and their respective efficacy and effectiveness in patients exposed to BTKi and venetoclax.

## 2 Methods

This study's protocol was registered in the International Prospective Register of Systematic Reviews (PROSPERO; CRD42023476609). This review followed the 2020 Preferred Reporting Items for Systematic Reviews and Meta‐analyses (PRISMA) reporting guideline [21].

### 2.1 Data Sources and Search Strategy

A comprehensive search was performed in PubMed, Embase, and Web of Science for the relevant studies from database inception to December 2023. To identify additional records, a gray literature search was performed using ClinicalTrials.gov, conference abstracts of the American Society of Clinical Oncology (ASCO) and European Society of Medical Oncology (ESMO) annual meetings, as well as the bibliographies of the selected studies were searched. The search strategy was developed using the terms “chronic lymphocytic leukemia,” “relapsed/refractory,” and “treatment outcomes.”

### 2.2 Study Selection

This systematic review included clinical trials and observational studies that met the following criteria: patients with CLL/SLL who had been exposed to both BTKi and BCL2 inhibitors; treated with any systemic therapy or chimeric antigen receptors (CAR)‐T therapy; reporting any efficacy outcomes (survival or best response); study designs such as randomized controlled trials, single‐arm trials, nonrandomized trials, and cohort studies. Two independent reviewers (M.Z. and S.A.) performed the screening of the title/abstract. The relevant full texts were further screened for the potentially eligible articles for inclusion in this study. Any disagreements were resolved by discussion with the third reviewer (L.V.Z).

### 2.3 Data Extraction

The variables extracted for this review include the study characteristics, patient characteristics, information on prior treatment, overall survival (OS), PFS, overall response rate (ORR), complete response (CR), partial response (PR), progressive disease (PD) and stable disease (SD). Risk of bias assessment was performed using the ROBINS‐I tool for interventional studies and the New Castle Ottawa scale (NOS) for observational studies [22, 23]. Two reviewers (M.Z. and S.A.) independently extracted the data and performed the quality appraisal. Any disagreements were resolved through the third reviewer (L.V.Z).

### 2.4 Data Analysis

We employed the narrative synthesis approach and presented the results in the tabular form. We explored the opportunity of performing the meta‐analysis. However, without data from randomized controlled and observational studies of comparative effectiveness, the meta‐analysis could not be performed.

## 3 Results

### 3.1 Study Selection

**FIGURE 1:**
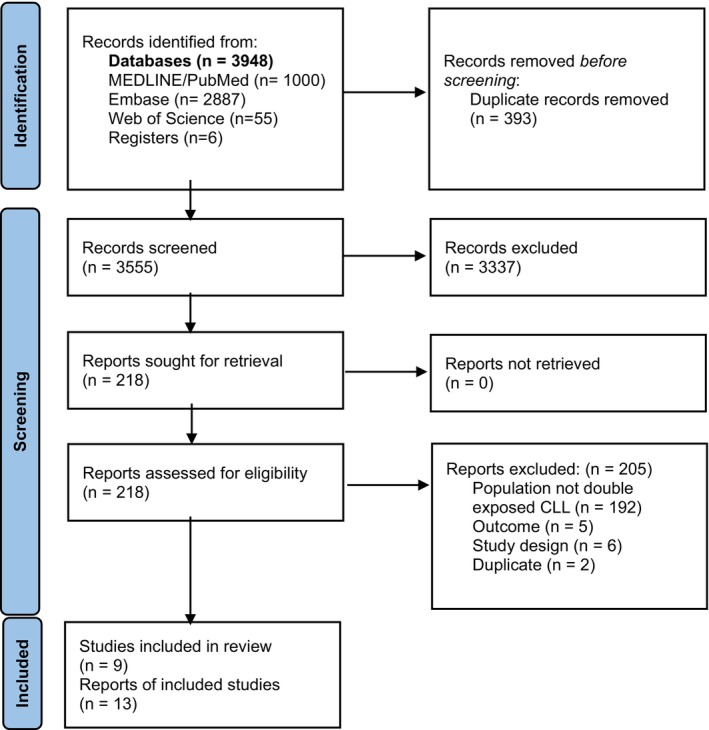
PRISMA flow diagram.

A total of 3948 records were identified through the databases and registers. After removing 393 duplicates, 3555 records were screened for eligibility and 3337 were excluded in title/abstract screening. We considered 218 records for full‐text screening, and we included 13 records reporting on nine studies in this systematic review [4, 24, 25, 26, 27, 28, 29, 30, 31]. Figure 1 illustrates the study selection process as per the PRISMA 2020 guidelines.

### 3.2 Study Characteristics

We included nine studies; five clinical trials, and four observational studies [4, 24, 25, 26, 27, 28, 29, 30, 31]. All five clinical trials were single‐arm trials, Mato et al., 2023 and Kater et al., 2023 were two multicenter clinical trials [24, 26]. Siddiqi et al., 2022 and Woyach et al., 2022 were conducted in the United States [28, 31]. Turtle et al., 2016 did not report the study location [30]. The clinical trials were published between 2016 and 2023 [24, 26, 28, 30, 31]. All four observational studies were retrospective in nature [4, 25, 27, 29]. Among observational studies, Thompson et al., 2021 and Mato et al., 2020 were multicenter international studies [4, 29]. In contrast, Hampel et al., 2022 and Din et al., 2023 were single‐center studies conducted in the United States [25, 27]. The retrospective studies were published from 2020 to 2023 [4, 25, 27, 29]. The detailed study characteristics are given in Table 1.

**TABLE 1:** Study characteristics.

Author, year	Study design	Study location	Data source	Study period	No. of patients analyzed in the study	No. of patients in double‐exposed setting
Mato et al., 2023 [26]	Single arm trail	Australia, France, Italy, Japan, Poland, South Korea, Sweden, Switzerland, United Kingdom, and United States	NA	March 21, 2019 to July 29, 2022	317	100
Siddiqi et al., 2022 [28]	Single arm trail	United States	NA	January 2018 to January 2019	23	11
Woyach et al., 2022 [31]	Single arm trail	United States	NA	NR	112	24
Turtle et al., 2016 [30]	Single arm trail	NR	NA	NR	18	4
Kater et al., 2023 [24]	Single arm trail	Australia, Belgium, Denmark, France, Germany, Israel, Italy, Netherlands, Spain, United Kingdom, United States	NA	NR	23	15
Thompson et al., 2021 [29]	Retrospective study	International	NR	NR	125	125
Hampel et al., 2022 [25]	Retrospective study	United States	Medical records Mayo Clinic	April 2012 to June 2021	33	11
Din et al., 2023 [27]	Retrospective study	United States	Medical records	2016 to 2021	17	17
Mato et al., 2020 [4]	Retrospective study	International (United States, European Union/United Kingdom, and South America)	Medical records	2014 to 2019	326	65

Abbreviations: NA, not applicable; NR, not reported.

### 3.3 Patient Characteristics

In the clinical trials, the number of double‐exposed CLL patients ranged from 4 to 100 [24, 26, 28, 30, 31]. All five clinical trials had double‐exposed CLL patients as a subgroup [24, 26, 28, 30, 31]. Woyach et al., 2022 and Kater et al., 2023 did not report the baseline characteristics for double‐exposed CLL [24, 31]. The median age of double‐exposed CLL patients ranged from 60 to 69 years [24, 26, 28, 30, 31]. Mato et al., 2023 reported a higher proportion (67%) of male patients [26]. Conversely, Siddiqi et al., 2022 had slightly more female patients (52%) [28]. Two clinical trials reported on the *TP53* mutation [26, 28]. Mato et al., 2023 had 38%, and Siddiqi et al., 2022 had 61% of patients with *TP53* mutations [26, 28]. In only one clinical trial, Mato et al., 2023 reported the *C481* mutations in 33% of patients [26]. Mato et al., 2023 also reported the *DEL(17p)* mutational status, finding that 30% of patients had *DEL(17p)* mutation [26]. Two clinical trials reported the IGHV unmutated and complex karyotype status [26, 28]. In Mato et al., 2023, 92% of patients had IGHV unmutated, and 37% of patients had complex karyotypes [26]. Siddiqi et al., 2022, reported 35% of patients with IGHV unmutated status and 48% of patients with complex karyotypes status [28].

The median lines of previous treatments ranged from 4 to 5 [24, 26, 28, 30, 31]. Only one clinical trial reported the reason for the BTKi discontinuation [26]. Mato et al., 2023, reported that 84% of patients discontinued the treatment due to PD, and 16% of patients discontinued due to adverse events or toxicity [26]. The reason for discontinuing the venetoclax was not reported in any clinical trials.

In observational studies, number of double‐exposed patients ranged from 11 to 326 [4, 25, 27, 29]. The median age ranged from 55.5 to 76 years [4, 25, 27, 29]. The proportion of male patients ranged from 41.2% to 69.6% [4, 25, 27, 29]. Two observational studies reported *TP53* mutations, 82% in Hampel et al., 2022, and 45% in Mato et al., 2020 [4, 25]. Two observational studies reported the *DEL(17p)* mutational status. Hampel et al., 2022 had 82% of patients with *DEL(17p)* mutation [25]. Whereas Din et al., 2023 had 64.7% of patients with *DEL(17p)* mutation [27]. No observational study has been reported on a number of patients with *C481* mutations. All four observational studies reported IGHV unmutated status ranging from 82% to 92.9% [4, 29]. Three studies reported complex karyotypes which ranged from 39% to 80% [4, 27]. The median line of previous treatments ranged from 3 to 4 [4, 25, 27, 29]. Only Thompson et al., 2021 reported the reason for BTKi and venetoclax discontinuation [29]. The reason for BTKi discontinuation was PD in 71.1% of patients and adverse events in 25.6% of patients [29]. Patients discontinued venetoclax due to PD in 68.8% of patients and due to adverse events in 16.87% of patients [29]. The detailed patient characteristics are given in Table 2.

**TABLE 2:** Patient characteristics.

Author, year	Treatment name	*N*	Age (median, range)	Gender, %		Mutational status					Prior treatments	Reason for BTKi discontinuation, %		Reason for VEN discontinuation, %	
				Male *N* (%)	Female *N* (%)	*TP53 N* (%)	*C481* mutation *N* (%)	*DEL(17p) N* (%)	*IGHV* unmutated *N* (%) %	Complex karyotype *N* (%)	Median lines of treatment (range)	AE/toxicity	PD	AE	PD
Clinical trials															
Mato et al., 2023 [26]	Pirtobrutinib	100	69 (41, 88)	67 (67)	33 (33)	34 (89)^a^	29 (33)	19 (30)	(92)	(37)	5 (1–11)	16	84	—	—
Woyach et al., 2022 [31]	Nemtabrutinib	—	—	—	—	—	—	—	—	—	—	—	—	—	—
Siddiqi et al., 2022 [28]	Lisocabtagene maraleucel	23	66 (50, 80)	11 (48)	12 (52)	14 (61)	—	8 (35)	(35)	(48)	4 (2–11)			—	—
Turtle et al., 2016 [30]	Anti‐CD19 CAR‐T cells	18	60 (40, 73)	—	—	—	—				5 (3–9)	—	—	—	—
Kater et al., 2023 [24]	Epcoritamab	—	—	—	—	—	—	—	—	—	—	—	—	—	—
Observational studies															
Hampel et al., 2022 [25]	Ibrutinib + venetoclax	11	76 (54, 86)	6 (54)	5 (46)	9 (82)	—	—	(90)	(80)	—	—	—	—	—
Din et al., 2023 [27]	CAR‐T Acalabrutinib + obinutuzumab	17	66 (47, 80)	7 (41.2)	10 (58.8)	—	—	11 (64.7)	(88.2)	(52.9)	3 (1–7)	—	—	—	—
Thompson et al., 2021 [29]	ncBTKi PI3Ki AlloSCT CAR T‐cell transplant CIT VEN re‐treatment cBTKi	125	55.5 (32, 83)	(69.6)	(30.4)	—	—	—	(92.9)	—	4 (2–12)	25.6	71.1	16.8	68.8
Mato et al., 2020 [4]	BTKi PI3Ki CAR‐T	326	58 (32, 88)	(69)	(31)	(45)	—	—	(82)	(39)	3 (0–11)	—	—	—	—

*Note:* —, not reported.

Abbreviations: AE, Adverse event; BTKi, Bruton's tyrosine kinase inhibitors; CAR‐T, chimeric antigen receptor T‐cell; cBTKi, covalent BTKi; CI, confidence intervals; CIT, chemoimmunotherapy; IGHV: immunoglobulin heavy chain variable region; *N*, number of patients; ncBTKI, noncovalent BTKi; PD, progressive disease; PIK3Ki, Phosphoinositide 3‐kinases inhibitors; VEN, venetoclax.

^a^ Total number of patients (*N*) assessed for *TP53* in Mato et al., 2023 is 38.

### 3.4 Efficacy/Effectiveness and Treatment Options in Double‐Exposed CLL

The treatments have been categorized into different classes for the ease of reporting the results. Nine treatment classes were used to treat double‐exposed CLL/SLL patients including noncovalent BTKi (ncBTKi), CAR‐T cell therapy, bispecific CD20‐directed CD3 T‐cell engager, BTKi, PI3Ki, allogeneic stem cell transplantation (AlloSCT), BCL2 inhibitor retreatment, CIT, and BTKi combination therapy. Table 3 provides detailed efficacy/effectiveness results for each treatment.

**TABLE 3:** Efficacy of treatments in double‐exposed CLL patients.

Author, year	Treatment name	Treatment class	*N*	Follow up	Survival outcomes		Response outcomes				
					PFS, median (95 CI)	OS, median (95 CI)	ORR, % (95 CI)	CR, %	PR, %	SD, %	PD, %
Clinical trials											
Mato et al., 2023 [26]	Pirtobrutinib	ncBTKi	100	18.2	16.8 (13.2, 18.7)	—	70.0 (60.0–78.8)	0	70	11	—
Mato et al., 2023 [26]	Pirtobrutinib		122	13.9	14.1 (11.1, 18.7)	—	73 (64–81)	—	—	—	—
Mato et al., 2023 [26]	Pirtobrutinib		54	6	—	—	64 (49–78)	—	—	—	—
Woyach et al., 2022 [31]	Nemtabrutinib		24	8.1	10.1 (7.4, 15.9)	—	58 (37–78)	0	25	—	—
Siddiqi et al., 2022 [28]	Lisocabtagene maraleucel	CAR‐T cell therapy	24	11	13 (2.8, NR)	—	80	60	20	20	0
Turtle et al., 2016 [30]	CAR‐T		4	8.4	—	—	—	—	50	—	—
Kater et al., 2023 [24]	Epcoritamab	Bispecific CD20‐directed CD3 T‐cell engager	15	9.3	—	—	53	27	—	—	—
Observational studies											
Thompson et al., 2021 [29]	ncBTKi	ncBTKi	45	9	—	—	75	—	—	—	—
Thompson et al., 2021 [29]	CAR‐T	CAR‐T cell therapy	9	3	4	—	85.7	—	—	—	—
Din et al., 2023 [27]	CAR‐T		6	—	—	—	—	50^a^	—	—	—
Mato et al., 2020 [4]	CAR‐T		18	2	9	—	66.6	33.3	33.3	5.7	27.7
Thompson et al., 2021 [29]	cBTKi	BTKi	43	—	—	—	53.7	—	—	—	—
Mato et al., 2020 [4]	BTKi		30	3.5	12	—	53.4	10	26.7	23.3	23.3
Thompson et al., 2021 [29]	PI3Ki	PI3Ki	24	4	5	—	40.9	—	—	—	—
Mato et al., 2020 [4]	PI3Ki		17	5	5	—	46.9	5.9	35.2	23.7	29.4
Hampel et al., 2022 [25]	Ibrutinib + venetoclax	BTKi combination retreatment	11	23.8	—	27 (15.5, NE)	100	55	45	—	—
Din et al., 2023 [27]	Acalabrutinib + obinutuzumab		2	—	—	—	—	50^a^	—	—	—
Thompson et al., 2021 [29]	VEN re‐treatment	BCL2i retreatment	6	—	14	—	40	—	—	—	—
Thompson et al., 2021 [29]	AlloSCT	AlloSCT	17	6.5	11	—	76.5	—	—	—	—
Thompson et al., 2021 [29]	CIT	CIT	23	2	3	—	31.8	—	—	—	—

*Note:* —, not reported.

Abbreviations: BCL2i, B‐cell lymphoma‐2 inhibitors; BTKi, Bruton's tyrosine kinase inhibitors; CAR‐T, chimeric antigen receptor T‐cell; cBTKi, covalent BTKi; CI, confidence intervals; CIT, chemoimmunotherapy; CR, complete response; *N*, number of patients; ncBTKI, noncovalent BTKi; NE, not evaluable; NR, not reached, OS, overall survival; PD, progressive disease; PFS, progression‐free survival; PIK3Ki, Phosphoinositide 3‐kinases inhibitors; PR, partial response; SD, stable disease; VEN, venetoclax.

^a^ Calculate‐not reported.

#### 3.4.1 Noncovalent BTK Inhibitors

We identified two clinical trials assessing the efficacy of ncBTKi, pirtobrutinib, and nemtabrutinib [26, 31]. Both clinical trials reported the median PFS. At a median follow‐up of 18.2 months Mato et al., 2023 reported the median PFS of 16.8 (95% CI, 13.2–18.7) months with pirtobrutinib [26]. Woyach et al., 2022 reported a median PFS of 10.1 (95% CI, 7.4–15.9) months at the 8.1‐month follow‐up for patients treated with nemtabrutinib [31]. Both the trials reported the ORR [26, 31]. The ORR in patients treated with pirtobrutinib was 70% (CR: 0%, PR: 70%) [26]. In patients treated with nemtabrutinib, the ORR was 58% (CR: 0, PR:25%) [31]. Neither ncBTK inhibitor study reported a CR [26, 31].

#### 3.4.2 CAR‐T Cell Therapy

Overall, five studies (two clinical trials and three observational studies) assessed the efficacy and effectiveness of CAR‐T cell therapy in double‐exposed CLL patients [4, 27, 28, 29, 30]. In clinical trials, Siddiqi et al., 2022, assessed the efficacy of lisocabtagene maraleucel [28]. At 11 months of follow‐up, the median PFS was 13 (95% CI, 2.8–not reached) months, and the ORR was seen in 80% (CR: 60%, PR: 20%) [28]. Another clinical trial by Turtle et al., 2016 showed that patients treated with anti‐CD19 CAR‐T cells had an ORR of 50% (CR:0, PR of 50%) [30].

All three observational studies reported response outcomes, but none of the studies reported survival outcomes for CAR‐T cell treatment [4, 27, 29]. At 3 months follow‐up, Thompson et al., 2021 reported the ORR in 85.7% of patients [29]. Din et al., 2023 reported CR in 50% of the patients [27]. Mato et al., 2020 reported an ORR of 66.6% (CR: 33.3%, PR: 33.3%) of patients [4].

#### 3.4.3 BTK Inhibitors Retreatment

Two retrospective studies, Thompson et al., 2021, and Mato et al., 2020 analyzed the effectiveness of BTKi [4, 29]. Thompson et al., 2021 reported a collective ORR of 53.7% in patients treated with BTKi, including ibrutinib, acalabrutinib, and noncovalent BTKi [29]. Mato et al., 2020 reported a PFS of 12 months and an ORR of 53.4% (CR: 10%, PR: 26.7) [4].

#### 3.4.4 PI3K Inhibitors

Thompson et al., 2021 and Mato et al., 2020 are the two observational studies that reported the effectiveness results for PI3K inhibitors [4, 29]. Thompson et al., 2021 reported a median PFS of 5 months and an ORR of 40.9% at 4 months follow‐up [29]. In a retrospective study by Mato et al., 2020, the median PFS was 5 months, and the ORR was 44.6% (CR: 5.9%, PR: 35.2%) [4].

#### 3.4.5 BTK Inhibitors Combination Retreatment

Two observational studies assessed the effectiveness of BTKi combination retreatment. Hampel et al., 2022 conducted a retrospective study to assess the effectiveness of ibrutinib with venetoclax combination retreatment in double‐exposed CLL patients. At a median follow‐up of 23.8 months, the study reported a median OS of 27 (95% CI, 15.5–not evaluable) months. The ORR was reported in all patients 100% (CR: 55%, PR: 45%) [25]. Another retrospective study by Din et al., 2023 assessed the effectiveness of acalabrutinib + obinutuzumab. Of two patients, one patient showed the objective response [27].

#### 3.4.6 BCL2 Retreatment

Thompson et al., 2021 conducted a study to assess the effectiveness of venetoclax retreatment. The study reported a median PFS of 14 months and an ORR in 40% of patients [29].

#### 3.4.7 Allogeneic Stem Cell Transplantation (AlloSCT)

Thompson et al., 2021 reported the PFS and ORR for AlloSCT. At 6.5 months of follow‐up, the median PFS was 11 months, and ORR was reported in 76.5% of patients [29].

#### 3.4.8 Chemoimmunotherapy

Thompson et al., 2021 was the only observational study to report the effectiveness of chemoimmunotherapy. At a median follow‐up of 2 months, the ORR was found to be 31.8% in double‐exposed patients [29].

#### 3.4.9 Bispecific CD20‐Directed CD3 T‐Cell Engager

Kater et al., 2023 reported the efficacy results for subcutaneous epcoritamab. At 9.3 months of follow‐up, the study reported 53% ORR (CR: 27%, PR: 26%) [24].

### 3.5 Quality Assessment

The quality assessment of included clinical trials using ROBINS‐I showed that three trials were at low risk of bias [26, 28, 30]. Woyach et al., 2022 and Kater et al., 2023 were the two clinical trials graded as moderate risk of bias [24, 31]. Two observational studies were graded as good quality and two as fair quality studies based on the NOS scale [4, 25, 27, 29]. The complete description of the quality assessment of studies is given in Appendix S1.

## 4 Discussion

Our systematic review highlights the complex treatment landscape for double‐exposed CLL/SLL patients who have previously received both BTKi and venetoclax. This patient cohort represents a particularly challenging group, given their poor prognosis and limited therapeutic options after standard treatments fail. The efficacy of emerging therapies like pirtobrutinib and CAR‐T cell therapies, including lisocabtagene maraleucel, introduces a potential shift in the treatment paradigm, providing hope for durable remissions in a population characterized by high relapse rates and significant treatment resistance.

Two narrative reviews have been published regarding the treatment approach and unmet clinical needs in double‐exposed CLL patients [18, 32]. To our knowledge, this is the first systematic review conducted on this topic. Our research identified pirtobrutinib as a promising treatment, demonstrating a better PFS of 16.8 months and ORR of 70% in clinical trial settings among double‐exposed CLL patients [20]. Lisocabtagene maraleucel, a CAR‐T cell therapy, showed a promising response with an ORR of 80% (CR: 60 and PR: 20) in clinical trials [28]. Additionally, a combination of ibrutinib and venetoclax achieved a median OS of 27 months and ORR of 100% (CR: 55% and PR: 45%) in a real‐world setting [25]. Given this evidence, pirtobrutinib, lisocabtagene maraleucel and combination with ibrutinib + venetoclax might be an effective treatment in double‐exposed CLL patients. Since the results are from the single‐arm/observational studies, the outcomes may be confounded by the patient characteristics.

All clinical trials included in the review were Phase I or II, and they generally had double‐exposed patients as a subgroup. Our study did not identify any Phase III studies with double‐exposed CLL patients. The number of patients included in the clinical trials ranged from 4 to 100, while the observational studies had sample sizes ranging from 11 to 125. Although these sample sizes are small, they are consistent with the nature of the disease and prior treatment exposure. In the future, where the use of BTKi and BCL2 inhibitors is higher in front‐line settings of CLL, larger sample sizes of double‐exposed patients can yield more reliable findings.

The review also identified studies with outcomes for double‐exposed CLL that did not report specific interventions; these studies were excluded from our systematic review. Eyre et al. conducted a real‐world study involving 215 patients who discontinued both covalent BTKi and BCL2 inhibitors. The study reported a median OS of 14.1 months following discontinuation and concluded that patients exposed to both BTKi and BCL2 inhibitors experienced poor outcomes [33]. Another retrospective study by Mato et al. analyzed data from 581 patients in the US who discontinued both BTKi and BCL2 inhibitors. The duration from discontinuation to the next line of therapy was 5.5 months, and the median time to either the next therapy discontinuation or death was 5.6 months [20]. Awan et al. conducted a retrospective study on 57 patients who received both BTKi and BCL2 inhibitors. The median time to the next treatment was 6.6 months, and the median OS was 16.6 months. The study also reported an OS rate of 57.5% at 12 months [34].

A study conducted by Samples et al. reported the effectiveness results for both double‐exposed and double‐refractory patients (i.e., those who progressed on both BTKi and BCL2 inhibitors). The median OS in the double‐exposed CLL cohort was 36.6 months (*n* = 66), whereas for the double‐refractory cohort, it was 21.2 months. The double‐exposed patients who started the next line of treatment had a median PFS of 8.4 months, while in the double‐refractory cohort, it was only 6.8 months [35].

The number of patients treated with novel agents, particularly BTKi and venetoclax, is steadily increasing. However, these patients often eventually discontinue their treatment or experience disease progression, with many developing resistance due to mutations [36]. Alarming, almost 50% of patients treated with both covalent BTKi and venetoclax will relapse on both drugs [35]. This significantly limits the options for novel treatments, leaving fewer therapeutic choices available. Patients with double refractory disease have poor health outcomes, characterized by significantly shorter PFS and OS [35]. Covalent BTK inhibitors exert their effect on the B‐cell receptor (BCR) by irreversibly binding to the *C481* residue of BTK, thereby inactivating the enzyme. The acquired cysteine‐to‐serine mutation at the BTK *C481* site confers resistance to these covalent BTKi [36]. A study conducted by Blombery et al., on 15 patients who progressed on venetoclax identified a *BCL2* single‐nucleotide variant, *G101V*, in seven patients. The presence of the *G101V* mutation significantly hindered venetoclax's ability to compete with BH3‐only proteins for binding to BCL2 [37, 38].

Emerging treatments are being developed to address the BTKi resistance in CLL patients, including “BTK‐degraders”, which target BTK for proteasomal degradation. These degraders are believed to reduce the risk of treatment resistance caused by BTK mutations [39]. Initial findings from a Phase I study of the selective BTK degrader NX‐5948 showed one PR among three evaluable patients [40]. Another Phase I trial assessed the safety and tolerability of BTK degrader Bgb‐16673 in 10 CLL patients, with five of six response‐evaluable patients responding to the treatment [41]. However, both studies do not specify whether these results pertain to double‐exposed CLL patients [40, 41].

Our systematic review has several limitations. First, the review identified only a few studies (*n* = 9) with smaller sample sizes, reflecting the scarcity of evidence available regarding treatments for double‐exposed patients. Second, we could not find any studies that specifically addressed double refractory patients. Also, data on the reasons for discontinuing BTKi and BCL2 inhibitors are missing in the included studies. Consequently, this study could only provide evidence for treatment results for double‐exposed patients irrespective of the reasons for discontinuation (whether due to progression or intolerance). Therefore, our results may not accurately reflect the outcomes for double refractory patients, where the reason for discontinuation is specifically progression. Finally, in our study, we could not perform a meta‐analysis due to different interventions, differences in study design, and the variety of outcomes reported in the studies we identified. The lack of overlapping interventions between studies also precludes conducting a network meta‐analysis.

We conducted a thorough literature search, scanning multiple databases and conference abstracts to identify all relevant studies, both clinical trials and observational studies. By including a wide range of study designs, we were able to capture a broad picture of the current evidence on this important topic. Additionally, we assessed the quality of the included studies using standardized tools, which helped contextualize the reliability of the findings.

## 5 Conclusion

Overall, the results of this systematic review indicate that there are limited data regarding the efficacy and effectiveness of treatments for double‐exposed CLL/SLL patients. In the identified clinical trials and real‐world studies, several treatments were tested including pirtobrutinib, lisocabtagene maraleucel, nemtabrutinib, CAR‐T cell, ncBTKi, epcoritamab, PI3K inhibitors, AlloSCT, chemoimmunotherapy, venetoclax retreatment, covalent BTKi, and combinations such as ibrutinib with venetoclax, and acalabrutinib with obinutuzumab. Of these, pirtobrutinib, lisocabtagene maraleucel, and the combination of ibrutinib and venetoclax have shown promising effects in these patients. However, the limited clinical data and available treatment options for patients who fail on BTKi and BCL2 inhibitors highlight a significant unmet need. Carefully designed clinical trials with balanced treatment groups should be commissioned to better understand the efficacy outcomes in double‐exposed CLL/SLL patients.

## Author Contributions

**Mohammed Zuber:** conceptualization (equal), data curation (equal), formal analysis (equal), writing – original draft (equal). **Sreelatha Akkala:** conceptualization (equal), data curation (equal), methodology (equal), resources (equal), writing – review and editing (equal). **Niying Li:** conceptualization (equal), writing – review and editing (equal). **Sajesh K. Veettil:** conceptualization (equal), writing – review and editing (equal). **Chia Jie Tan:** writing – review and editing (equal). **Lorenzo Villa Zapata:** conceptualization (equal), methodology (equal), writing – review and editing (equal).

## Ethics Statement

The authors have nothing to report.

## Conflicts of Interest

The authors declare no conflicts of interest.

## Supporting information


Appendix S1.

## Data Availability

Dr. Lorenzo Villa Zapata had full access to all the data in the study and took responsibility for the integrity of the data and the accuracy of the data analysis. Data were extracted from published clinical trials and observational studies, all of which are available and accessible.
