# Supplementary material for: Efficacy and Effectiveness Outcomes of Treatments for Double‐Exposed Chronic Lymphocytic Leukemia and Small Lymphocytic Lymphoma Patients: A Systematic Literature Review
Source: Cancer Med. 2024 Sep 30;13(18):e70258. doi: 10.1002/cam4.70258 (PMC11440144; doi:10.1002/cam4.70258)
Supplement: Supplementary file 1 — Appendix S1. [file CAM4-13-e70258-s001.docx]

**Appendix 1A: Quality Assessment of Clinical Trials**

| **Author, year** | **D1** | **D2** | **D3** | **D4** | **D5** | **D6** | **D7** | **D8** |
| --- | --- | --- | --- | --- | --- | --- | --- | --- |
| Mato et al., 2023 | Low | Low | Low | Low | Low | Low | Low | Low |
| Siddiqi et al., 2022 | Low | Low | Low | Low | Moderate | Low | Low | Low |
| Woyach et al., 2022 | Moderate | Low | Low | Low | Moderate | Moderate | Low | Moderate |
| Turtle et al., 2016 | Low | Low | Low | Low | Moderate | Moderate | Low | Low |
| Kater et al., 2023 | Moderate | Low | Low | Moderate | Moderate | Moderate | Low | Moderate |

D: Domain

**Appendix 1B: Quality Assessment of Observational Studies**

| **NOS criteria** | **Studies (year)** | | | |
| --- | --- | --- | --- | --- |
|  | Thompson et al., 2021 | Hampel et al., 2022 | Din et al., 2023 | Mato et al., 2020 |
| A. Selection (maximum of four stars) |  |  |  |  |
| 1. Representativeness of the exposed cohort | ★ | ★ | ★ | ★ |
| 2. Selection of the non-exposed cohort | ☆ | ☆ | ★ | ☆ |
| 3. Ascertainment of exposure | ★ | ★ | ★ | ★ |
| 4. Demonstration that outcome of interest was not present at start of study | ★ | ☆ | ☆ | ★ |
| B. Comparability (maximum of two stars) |  |  |  |  |
| 1. Comparability of cohort on the basis of the design or analysis | ★★ | ★☆ | ★☆ | ★☆ |
| C. Outcome (maximum of three stars) |  |  |  |  |
| 1. Assessment of outcome | ★ | ★ | ★ | ★ |
| 2. Was follow-up long enough for outcomes to occur | ★ | ★ | ☆ | ★ |
| 3. Adequacy of follow-up of cohorts | ☆ | ☆ | ☆ | ☆ |
| Total (maximum of nine stars) | 7 | 5 | 5 | 6 |
